# {2-[(1,3-Benzo­thia­zol-2-yl)meth­oxy]-5-fluoro­phen­yl}(4-chloro­phen­yl)methanone

**DOI:** 10.1107/S1600536813014621

**Published:** 2013-06-08

**Authors:** K. N. Venugopala, Susanta K. Nayak, Thavendran Govender, Hendrik G. Kruger, Glenn E. M. Maguire

**Affiliations:** a School of Pharmacy and Pharmacology, University of KwaZulu-Natal, Durban 4000, South Africa; b Equipe Chimie du Solide et Matériaux, UMR 6226 Institut des Sciences, Université de Rennes 1, Campus de Beaulieu, Avenue du Général Leclerc, 35042 Rennes Cedex, France; c School of Chemistry and Physics, University of KwaZulu-Natal, Durban 4000, South Africa

## Abstract

The asymmetric unit of the title compound, C_21_H_13_ClFNO_2_S, contains two independent mol­ecules with similar conformations. In the mol­ecules, the thia­zole ring is essentially planar [maximum atomic deviations = 0.014 (4) and 0.023 (5) Å] and is oriented with respect to the fluoro­phenyl ring and chloro­phenyl rings at 9.96 (18) and 70.39 (18)° in one mol­ecule and at 7.50 (18) and 68.43 (18)° in the other; the dihedral angles between the fluoro­phenyl and chloro­phenyl rings are 64.9 (2) and 64.6 (2)°, respectively. Inter­molecular C—H⋯O and C—H⋯F hydrogen bonds stabilize the three-dimensional supra­molecular architecture. Weak C—H⋯π and π–π inter­actions [centroid–centroid distance = 3.877 (3) Å] lead to a criss-cross mol­ecular packing along the *c* axis.

## Related literature

For background to the applications of benzo­thia­zole derivatives, see: Rana *et al.* (2007 ▶); Saeed *et al.* (2010 ▶); Telvekar *et al.* (2012 ▶); Kelarev *et al.* (2003 ▶). For crystal structures of related benzo­thia­zoles, see: Nayak *et al.* (2013 ▶); Venugopala *et al.* (2012 ▶).
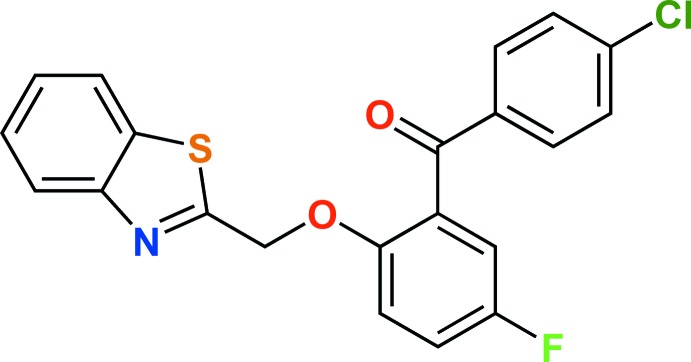


## Experimental

### 

#### Crystal data

C_21_H_13_ClFNO_2_S
*M* *_r_* = 397.84
Orthorhombic, 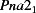

*a* = 19.7280 (6) Å
*b* = 7.4755 (3) Å
*c* = 24.4847 (7) Å
*V* = 3611.0 (2) Å^3^
*Z* = 8
Mo *K*α radiation
μ = 0.35 mm^−1^
*T* = 292 K
0.18 × 0.12 × 0.08 mm

#### Data collection

Oxford Diffraction Xcalibur (Eos, Nova) diffractometer
Absorption correction: multi-scan (*CrysAlis PRO*; Oxford Diffraction, 2009 ▶) *T*_min_ = 0.939, *T*_max_ = 0.972
36561 measured reflections
7100 independent reflections
4182 reflections with *I* > 2σ(*I*)
*R*_int_ = 0.076

#### Refinement

*R*[*F* ^2^ > 2σ(*F* ^2^)] = 0.053
*wR*(*F* ^2^) = 0.118
*S* = 0.99
7100 reflections
488 parameters
1 restraint
H-atom parameters constrained
Δρ_max_ = 0.31 e Å^−3^
Δρ_min_ = −0.21 e Å^−3^
Absolute structure: Flack (1983 ▶)
Flack parameter: −0.05 (8), **???? Friedel pairs**

### 

Data collection: *CrysAlis PRO* (Oxford Diffraction, 2009 ▶); cell refinement: *CrysAlis PRO*; data reduction: *CrysAlis PRO*; program(s) used to solve structure: *SHELXS97* (Sheldrick, 2008 ▶); program(s) used to refine structure: *SHELXL97* (Sheldrick, 2008 ▶); molecular graphics: *ORTEP-3 for Windows* (Farrugia, 2012 ▶) and *Mercury* (Macrae *et al.*, 2008 ▶); software used to prepare material for publication: *PLATON* (Spek, 2009 ▶) and *PARST* (Nardelli, 1995 ▶).

## Supplementary Material

Crystal structure: contains datablock(s) global, I. DOI: 10.1107/S1600536813014621/xu5707sup1.cif

Structure factors: contains datablock(s) I. DOI: 10.1107/S1600536813014621/xu5707Isup2.hkl

Supplementary material file. DOI: 10.1107/S1600536813014621/xu5707Isup3.cml

Additional supplementary materials:  crystallographic information; 3D view; checkCIF report

## Figures and Tables

**Table 1:** Hydrogen-bond geometry (Å, °) *Cg*1 and *Cg*2 are the centroids of the thia­zole rings S1/C1/C6/N1/C7 and S2/C22/C27/N2/C28, respectively.

*D*—H⋯*A*	*D*—H	H⋯*A*	*D*⋯*A*	*D*—H⋯*A*
C3—H3⋯F1^i^	0.93	2.52	3.091 (6)	120
C5—H5⋯O2^ii^	0.93	2.46	3.340 (5)	158
C26—H26⋯O4^iii^	0.93	2.51	3.369 (5)	154
C18—H18⋯*Cg*1^iv^	0.93	2.83	3.686 (5)	154
C39—H39⋯*Cg*2^v^	0.93	2.82	3.619 (5)	145

Symmetry codes: (i) 
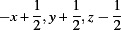
; (ii) 
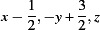
; (iii) 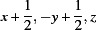; (iv) 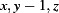; (v) 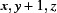.

**Fig. 1.**
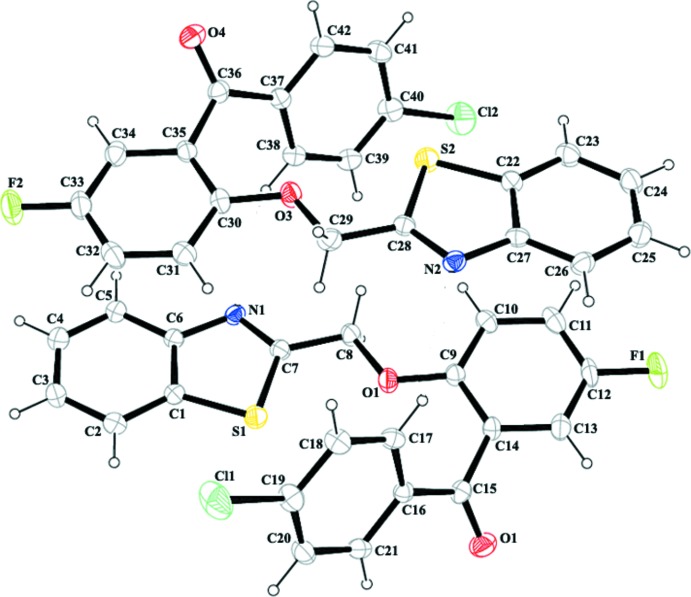
Molecular structure shows the atom labelling scheme with displacement ellipsoids for non-H atoms at 30% probability level, hydrogen atoms are arbitrary circle.

**Fig. 2.**
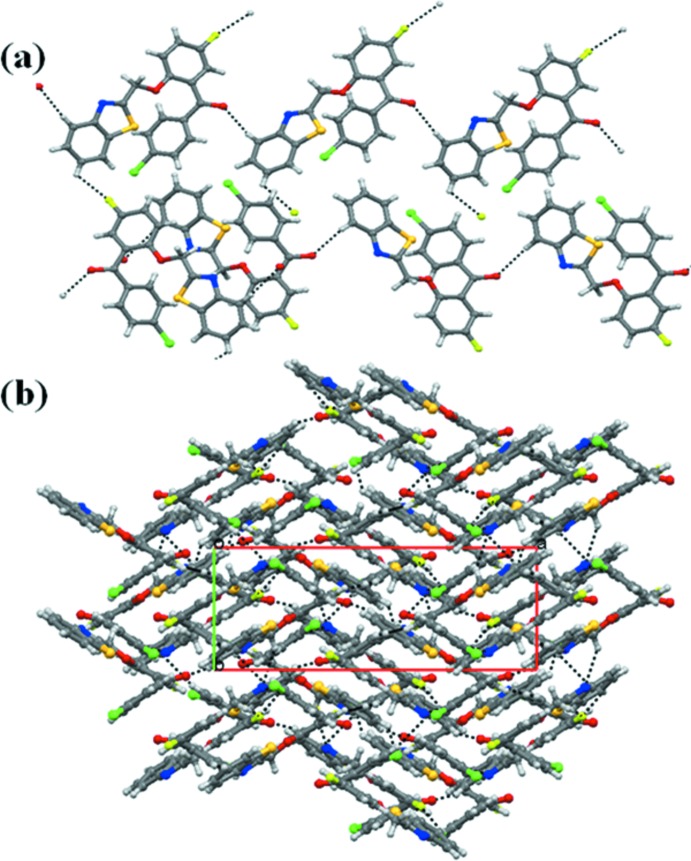
(a)The C—H···O and C—H···F hydrogen bond chains. (b) additional C—H···π and π···π interactions lead to criss-cross molecular assembly along c axis.

## supplementary crystallographic information

### Comment

Substituted benzothiazole derivatives exhibit various pharmacological properties such as analgesic, antimicrobial, antidepressant, antitumor, antihypertensive, anthelmintic, and herbicidal activity (Kelarev *et al.*, 2003). Thus the biological features of new benzothiazole derivatives is of great scientific interest (Telvekar *et al.*, 2012; Saeed *et al.*, 2010; Rana *et al.*, 2007). Several crystal structures of the benzothiazoles have been reported (Nayak *et al.*, 2013; Venugopala *et al.*, 2012). Here, we report the single-crystal structure of the title compound.

The title compound, C_21_H_13_ClFNO_2_S, prefers two symmetry independent molecules in the asymmetric unit (Fig. 1). The conformation of the individual molecules adopt the dihedral angles of 64.8 (2)° and 66.6 (2)° between the planes of their respective benzothiazole and chlorophenylmethanone groups. Intermolecular C—H···O and C—H···F hydrogen bond chains stabilize the three dimension molecular assembly. Further, the C—H···π [2.83 Å, *Cg*1 = Centroid of five membered ring S1/C1/C6/N1/C7; 2.82 Å, *Cg*2 = Centroid of five membered ring S2/C22/C27/N2/C28] and π···π [*Cg*3···*Cg*4 = 3.877 (3) Å, *Cg*3 = Centroid of six membered ring C16—C21 and *Cg*4 = Centroid of six membered ring C30—C35] interactions lead to criss-cross molecular packing along the *c* axis.

### Experimental

To a solution of (2-chloromethyl)benzo[*d*]thiazole (1 mmol) and (4-chlorophenyl)(5-fluoro-2-hydroxyphenyl)methanone (1 mmol) in dry THF, dry potassium carbonate (1 mmol) was added and stirred at room temperature. The reaction mixture was added and the reaction mixture was stirred at room temperature for 14 h. The reaction mixture was concentrated to remove the solvent, diluted with ethyl acetate, washed with water, brine solution and dried over anhydrous sodium sulfate. The organic layer was concentrated to yield a residue which was purified by column chromatography using ethyl acetate and n-hexane as eluent (7:3, Rf = 0.70) to afford the product in 78% as a white solid (m. p. 419 (2) K). Suitable crystals for single-crystal X-ray study were obtained from acetone solvent using slow evaporation technique at room temperature.

### Refinement

All H atoms were positioned geometrically and refined using a riding model with *U*_iso_(H)= 1.2 *U*_eq_(C).

### Crystal data

**Table:**

C_21_H_13_ClFNO_2_S	*D*_x_ = 1.464 Mg m^−^^3^
*M**_r_* = 397.84	Melting point: 419(2) K
Orthorhombic, *P**n**a*2_1_	Mo *K*α radiation, λ = 0.7107 Å
Hall symbol: P 2c -2n	Cell parameters from 340 reflections
*a* = 19.7280 (6) Å	θ = 2.7–27.0°
*b* = 7.4755 (3) Å	µ = 0.35 mm^−^^1^
*c* = 24.4847 (7) Å	*T* = 292 K
*V* = 3611.0 (2) Å^3^	Block, colorless
*Z* = 8	0.18 × 0.12 × 0.08 mm
*F*(000) = 1632	

### Data collection

**Table:**

Oxford Diffraction Xcalibur (Eos, Nova) diffractometer	7100 independent reflections
Radiation source: Mova (Mo) X-ray Source	4182 reflections with *I* > 2σ(*I*)
Mirror monochromator	*R*_int_ = 0.076
Detector resolution: 16.0839 pixels mm^-1^	θ_max_ = 26.0°, θ_min_ = 2.7°
ω scans	*h* = −24→24
Absorption correction: multi-scan (*CrysAlis PRO*; Oxford Diffraction, 2009)	*k* = −9→9
*T*_min_ = 0.939, *T*_max_ = 0.972	*l* = −30→30
36561 measured reflections	

### Refinement

**Table:**

Refinement on *F*^2^	Hydrogen site location: inferred from neighbouring sites
Least-squares matrix: full	H-atom parameters constrained
*R*[*F*^2^ > 2σ(*F*^2^)] = 0.053	*w* = 1/[σ^2^(*F*_o_^2^) + (0.037*P*)^2^] where *P* = (*F*_o_^2^ + 2*F*_c_^2^)/3
*wR*(*F*^2^) = 0.118	(Δ/σ)_max_ < 0.001
*S* = 0.99	Δρ_max_ = 0.31 e Å^−^^3^
7100 reflections	Δρ_min_ = −0.21 e Å^−^^3^
488 parameters	Extinction correction: *SHELXL97* (Sheldrick, 2008), Fc^*^=kFc[1+0.001xFc^2^λ^3^/sin(2θ)]^-1/4^
1 restraint	Extinction coefficient: 0.0012 (2)
Primary atom site location: structure-invariant direct methods	Absolute structure: Flack (1983)
Secondary atom site location: difference Fourier map	Flack parameter: −0.05 (8)

### Special details

**Table:**

Geometry. All s.u.'s (except the s.u. in the dihedral angle between two l.s. planes) are estimated using the full covariance matrix. The cell s.u.'s are taken into account individually in the estimation of s.u.'s in distances, angles and torsion angles; correlations between s.u.'s in cell parameters are only used when they are defined by crystal symmetry. An approximate (isotropic) treatment of cell s.u.'s is used for estimating s.u.'s involving l.s. planes.
Refinement. Refinement of *F*^2^ against ALL reflections. The weighted *R*-factor *wR* and goodness of fit *S* are based on *F*^2^, conventional *R*-factors *R* are based on *F*, with *F* set to zero for negative *F*^2^. The threshold expression of *F*^2^ > 2σ(*F*^2^) is used only for calculating *R*-factors(gt) *etc*. and is not relevant to the choice of reflections for refinement. *R*-factors based on *F*^2^ are statistically about twice as large as those based on *F*, and *R*- factors based on ALL data will be even larger.

### Fractional atomic coordinates and isotropic or equivalent isotropic displacement parameters (Å^2^)

**Table:**

	*x*	*y*	*z*	*U*_iso_*/*U*_eq_	
S2	0.06104 (6)	0.33720 (18)	0.47599 (5)	0.0583 (4)	
S1	0.17610 (6)	0.63708 (17)	0.25881 (5)	0.0595 (4)	
Cl2	0.06464 (8)	0.7998 (2)	0.57593 (6)	0.1029 (6)	
Cl1	0.18806 (8)	0.1307 (2)	0.15892 (6)	0.1061 (6)	
O1	0.22799 (14)	0.5887 (4)	0.36472 (11)	0.0561 (8)	
N1	0.08765 (16)	0.8291 (5)	0.31021 (13)	0.0426 (9)	
O2	0.41192 (16)	0.4498 (5)	0.33659 (13)	0.0686 (10)	
O3	0.00885 (14)	0.3921 (4)	0.37068 (11)	0.0537 (8)	
N2	0.14867 (17)	0.1432 (5)	0.42445 (14)	0.0442 (9)	
F1	0.37852 (17)	0.3630 (5)	0.53847 (12)	0.1056 (12)	
F2	−0.14027 (16)	0.6181 (5)	0.19584 (12)	0.0965 (11)	
C35	−0.0842 (2)	0.5483 (6)	0.33474 (16)	0.0444 (11)	
C6	0.06876 (18)	0.8298 (5)	0.25537 (18)	0.0420 (10)	
C27	0.1675 (2)	0.1403 (6)	0.47895 (18)	0.0427 (10)	
C28	0.0952 (2)	0.2386 (5)	0.41793 (18)	0.0406 (10)	
C16	0.3100 (2)	0.3434 (5)	0.29917 (17)	0.0397 (10)	
C37	−0.0700 (2)	0.6470 (6)	0.43582 (17)	0.0443 (11)	
C24	0.1953 (2)	0.1670 (7)	0.5891 (2)	0.0622 (14)	
H24	0.2059	0.1769	0.6260	0.075*	
C18	0.2180 (2)	0.1664 (6)	0.26527 (19)	0.0530 (12)	
H18	0.1816	0.0900	0.2717	0.064*	
C26	0.2223 (2)	0.0503 (6)	0.50034 (18)	0.0532 (12)	
H26	0.2501	−0.0184	0.4779	0.064*	
C34	−0.1231 (2)	0.6039 (6)	0.29010 (19)	0.0553 (13)	
H34	−0.1627	0.6688	0.2955	0.066*	
C9	0.2631 (2)	0.5326 (6)	0.40965 (16)	0.0446 (11)	
C1	0.1111 (2)	0.7311 (6)	0.22139 (19)	0.0492 (12)	
C19	0.2358 (3)	0.2103 (7)	0.21246 (19)	0.0571 (13)	
C38	−0.0117 (2)	0.7485 (5)	0.4272 (2)	0.0473 (12)	
H38	0.0004	0.7809	0.3918	0.057*	
C15	0.3522 (2)	0.4139 (6)	0.34429 (18)	0.0472 (11)	
C21	0.3291 (2)	0.3796 (6)	0.24532 (17)	0.0500 (12)	
H21	0.3673	0.4487	0.2384	0.060*	
O4	−0.17413 (16)	0.5686 (5)	0.39716 (13)	0.0701 (10)	
C17	0.2540 (2)	0.2356 (6)	0.30809 (19)	0.0461 (12)	
H17	0.2406	0.2100	0.3437	0.055*	
C40	0.0101 (3)	0.7524 (7)	0.5223 (2)	0.0609 (14)	
C2	0.0976 (2)	0.7182 (7)	0.1656 (2)	0.0652 (14)	
H2	0.1258	0.6527	0.1427	0.078*	
C10	0.2421 (2)	0.5674 (6)	0.46288 (17)	0.0515 (12)	
H10	0.2019	0.6291	0.4693	0.062*	
C41	−0.0482 (2)	0.6569 (7)	0.53269 (19)	0.0623 (14)	
H41	−0.0604	0.6271	0.5682	0.075*	
C32	−0.0457 (3)	0.4639 (7)	0.22913 (18)	0.0631 (14)	
H32	−0.0333	0.4358	0.1935	0.076*	
C29	0.0633 (2)	0.2694 (6)	0.36384 (19)	0.0507 (12)	
H29A	0.0964	0.3179	0.3385	0.061*	
H29B	0.0465	0.1574	0.3491	0.061*	
C14	0.3237 (2)	0.4423 (6)	0.39979 (17)	0.0426 (11)	
C5	0.0125 (2)	0.9163 (6)	0.23477 (18)	0.0535 (12)	
H5	−0.0158	0.9825	0.2575	0.064*	
C31	−0.0069 (2)	0.4062 (6)	0.27210 (17)	0.0546 (13)	
H31	0.0321	0.3393	0.2658	0.066*	
C22	0.1258 (2)	0.2402 (6)	0.51368 (18)	0.0453 (12)	
C42	−0.0883 (2)	0.6062 (7)	0.48870 (19)	0.0585 (13)	
H42	−0.1282	0.5435	0.4950	0.070*	
C11	0.2812 (3)	0.5095 (6)	0.50601 (18)	0.0633 (14)	
H11	0.2674	0.5295	0.5418	0.076*	
C36	−0.1134 (2)	0.5866 (6)	0.39001 (17)	0.0488 (11)	
C33	−0.1023 (3)	0.5621 (7)	0.2386 (2)	0.0635 (14)	
C39	0.0280 (2)	0.8013 (6)	0.4703 (2)	0.0546 (12)	
H39	0.0667	0.8696	0.4644	0.066*	
C8	0.1729 (2)	0.7062 (6)	0.37194 (18)	0.0504 (12)	
H8A	0.1884	0.8193	0.3868	0.061*	
H8B	0.1401	0.6548	0.3969	0.061*	
C30	−0.0263 (2)	0.4483 (6)	0.32504 (18)	0.0458 (11)	
C4	−0.0004 (2)	0.9022 (7)	0.1803 (2)	0.0667 (15)	
H4	−0.0384	0.9592	0.1659	0.080*	
C7	0.1416 (2)	0.7339 (6)	0.31678 (18)	0.0444 (11)	
C25	0.2354 (2)	0.0638 (7)	0.55564 (19)	0.0610 (13)	
H25	0.2720	0.0021	0.5705	0.073*	
C20	0.2919 (2)	0.3138 (6)	0.20268 (19)	0.0574 (13)	
H20	0.3046	0.3394	0.1670	0.069*	
C12	0.3407 (3)	0.4220 (7)	0.49521 (19)	0.0641 (14)	
C13	0.3629 (2)	0.3886 (6)	0.4435 (2)	0.0575 (13)	
H13	0.4039	0.3304	0.4378	0.069*	
C23	0.1399 (2)	0.2553 (6)	0.5689 (2)	0.0595 (13)	
H23	0.1124	0.3236	0.5917	0.071*	
C3	0.0414 (2)	0.8049 (8)	0.1455 (2)	0.0702 (16)	
H3	0.0314	0.7985	0.1085	0.084*	

### Atomic displacement parameters (Å^2^)

**Table:**

	*U*^11^	*U*^22^	*U*^33^	*U*^12^	*U*^13^	*U*^23^
S2	0.0601 (8)	0.0648 (8)	0.0499 (7)	0.0205 (7)	0.0044 (6)	−0.0056 (7)
S1	0.0573 (7)	0.0733 (9)	0.0478 (7)	0.0237 (6)	0.0023 (6)	−0.0068 (7)
Cl2	0.1140 (12)	0.1242 (14)	0.0707 (10)	−0.0212 (11)	−0.0177 (9)	−0.0367 (10)
Cl1	0.1281 (13)	0.1298 (15)	0.0602 (10)	−0.0478 (11)	−0.0180 (9)	−0.0170 (10)
O1	0.0629 (19)	0.067 (2)	0.0387 (17)	0.0271 (17)	−0.0047 (15)	−0.0040 (16)
N1	0.0363 (19)	0.045 (2)	0.047 (2)	0.0055 (17)	0.0011 (16)	−0.0016 (17)
O2	0.045 (2)	0.088 (3)	0.072 (2)	−0.0047 (19)	0.0011 (18)	−0.006 (2)
O3	0.0607 (19)	0.060 (2)	0.0403 (17)	0.0167 (17)	−0.0029 (15)	−0.0032 (16)
N2	0.0402 (19)	0.048 (2)	0.044 (2)	0.0019 (18)	0.0020 (17)	−0.0069 (18)
F1	0.113 (2)	0.145 (3)	0.0588 (19)	0.009 (2)	−0.0385 (18)	0.025 (2)
F2	0.102 (2)	0.122 (3)	0.065 (2)	−0.007 (2)	−0.0297 (17)	0.033 (2)
C35	0.045 (3)	0.047 (3)	0.041 (3)	−0.011 (2)	−0.004 (2)	0.006 (2)
C6	0.041 (2)	0.043 (3)	0.043 (3)	−0.001 (2)	0.001 (2)	0.003 (2)
C27	0.044 (2)	0.043 (3)	0.041 (3)	0.000 (2)	0.008 (2)	−0.007 (2)
C28	0.043 (2)	0.041 (3)	0.038 (3)	0.000 (2)	0.003 (2)	−0.0030 (19)
C16	0.039 (2)	0.036 (2)	0.044 (2)	0.004 (2)	0.001 (2)	0.000 (2)
C37	0.050 (3)	0.041 (3)	0.042 (3)	0.007 (2)	0.003 (2)	−0.003 (2)
C24	0.071 (3)	0.071 (4)	0.044 (3)	0.001 (3)	−0.004 (3)	0.003 (3)
C18	0.061 (3)	0.043 (3)	0.055 (3)	−0.013 (2)	0.002 (2)	−0.004 (2)
C26	0.044 (3)	0.061 (3)	0.055 (3)	0.007 (2)	0.003 (2)	−0.005 (2)
C34	0.052 (3)	0.054 (3)	0.060 (3)	−0.007 (2)	−0.010 (2)	0.011 (3)
C9	0.047 (3)	0.046 (3)	0.041 (3)	0.002 (2)	−0.004 (2)	−0.001 (2)
C1	0.043 (2)	0.058 (3)	0.046 (3)	0.010 (2)	0.001 (2)	0.004 (2)
C19	0.073 (3)	0.051 (3)	0.047 (3)	−0.007 (3)	−0.006 (3)	−0.012 (2)
C38	0.051 (3)	0.039 (3)	0.052 (3)	−0.002 (2)	0.006 (2)	−0.002 (2)
C15	0.046 (3)	0.041 (3)	0.054 (3)	0.004 (2)	−0.006 (2)	0.001 (2)
C21	0.042 (2)	0.059 (3)	0.049 (3)	−0.001 (2)	0.009 (2)	0.000 (2)
O4	0.046 (2)	0.095 (3)	0.069 (2)	−0.002 (2)	0.0001 (17)	−0.003 (2)
C17	0.050 (3)	0.042 (3)	0.046 (3)	0.001 (2)	0.008 (2)	−0.003 (2)
C40	0.068 (3)	0.062 (3)	0.053 (3)	−0.003 (3)	0.004 (3)	−0.022 (3)
C2	0.053 (3)	0.096 (4)	0.046 (3)	0.002 (3)	0.005 (3)	−0.001 (3)
C10	0.062 (3)	0.054 (3)	0.038 (3)	−0.002 (3)	−0.001 (2)	−0.005 (2)
C41	0.067 (3)	0.074 (4)	0.045 (3)	0.006 (3)	0.013 (3)	−0.008 (3)
C32	0.079 (4)	0.070 (4)	0.040 (3)	−0.021 (3)	−0.005 (3)	0.004 (3)
C29	0.049 (3)	0.054 (3)	0.050 (3)	0.007 (2)	0.006 (2)	−0.011 (2)
C14	0.047 (3)	0.043 (3)	0.038 (2)	0.002 (2)	−0.010 (2)	−0.001 (2)
C5	0.046 (3)	0.063 (3)	0.052 (3)	0.005 (2)	−0.002 (2)	0.007 (2)
C31	0.060 (3)	0.056 (3)	0.048 (3)	−0.003 (2)	0.002 (2)	−0.001 (2)
C22	0.048 (2)	0.046 (3)	0.042 (3)	0.001 (2)	0.001 (2)	0.002 (2)
C42	0.052 (3)	0.066 (3)	0.058 (3)	0.000 (3)	0.009 (2)	0.005 (3)
C11	0.084 (4)	0.067 (4)	0.039 (3)	−0.019 (3)	−0.008 (3)	0.005 (3)
C36	0.046 (3)	0.044 (3)	0.056 (3)	0.005 (2)	−0.002 (2)	0.006 (2)
C33	0.068 (3)	0.068 (4)	0.055 (3)	−0.011 (3)	−0.018 (3)	0.015 (3)
C39	0.053 (3)	0.048 (3)	0.063 (3)	−0.007 (2)	0.006 (3)	−0.008 (3)
C8	0.056 (3)	0.052 (3)	0.043 (3)	0.020 (2)	0.000 (2)	0.002 (2)
C30	0.057 (3)	0.040 (3)	0.041 (3)	−0.003 (2)	−0.008 (2)	0.003 (2)
C4	0.052 (3)	0.085 (4)	0.063 (3)	0.007 (3)	−0.003 (3)	0.020 (3)
C7	0.046 (2)	0.046 (3)	0.041 (3)	0.003 (2)	0.003 (2)	0.001 (2)
C25	0.056 (3)	0.069 (4)	0.058 (3)	0.009 (3)	−0.007 (2)	−0.001 (3)
C20	0.067 (3)	0.064 (4)	0.041 (3)	0.005 (3)	0.003 (2)	0.001 (2)
C12	0.075 (4)	0.072 (4)	0.045 (3)	−0.002 (3)	−0.025 (3)	0.018 (3)
C13	0.057 (3)	0.060 (3)	0.056 (3)	0.004 (3)	−0.013 (2)	0.010 (3)
C23	0.067 (3)	0.067 (4)	0.044 (3)	0.009 (3)	0.008 (3)	−0.004 (3)
C3	0.059 (3)	0.107 (5)	0.045 (3)	−0.005 (3)	−0.007 (3)	0.014 (3)

### Geometric parameters (Å, º)

**Table:**

S2—C22	1.735 (4)	C1—C2	1.395 (6)
S2—C28	1.737 (4)	C19—C20	1.371 (6)
S1—C1	1.726 (4)	C38—C39	1.373 (6)
S1—C7	1.733 (4)	C38—H38	0.9300
Cl2—C40	1.734 (5)	C15—C14	1.486 (6)
Cl1—C19	1.721 (5)	C21—C20	1.368 (6)
O1—C9	1.366 (5)	C21—H21	0.9300
O1—C8	1.408 (4)	O4—C36	1.218 (5)
N1—C7	1.291 (5)	C17—H17	0.9300
N1—C6	1.393 (5)	C40—C39	1.369 (7)
O2—C15	1.224 (5)	C40—C41	1.378 (6)
O3—C30	1.380 (5)	C2—C3	1.374 (6)
O3—C29	1.422 (5)	C2—H2	0.9300
N2—C28	1.283 (5)	C10—C11	1.378 (6)
N2—C27	1.386 (5)	C10—H10	0.9300
F1—C12	1.369 (5)	C41—C42	1.389 (6)
F2—C33	1.354 (5)	C41—H41	0.9300
C35—C30	1.387 (6)	C32—C33	1.356 (7)
C35—C34	1.398 (5)	C32—C31	1.371 (6)
C35—C36	1.498 (6)	C32—H32	0.9300
C6—C5	1.380 (5)	C29—H29A	0.9700
C6—C1	1.391 (6)	C29—H29B	0.9700
C27—C26	1.376 (5)	C14—C13	1.381 (5)
C27—C22	1.399 (5)	C5—C4	1.363 (6)
C28—C29	1.485 (6)	C5—H5	0.9300
C16—C17	1.385 (5)	C31—C30	1.388 (5)
C16—C21	1.398 (6)	C31—H31	0.9300
C16—C15	1.480 (6)	C22—C23	1.386 (6)
C37—C42	1.378 (6)	C42—H42	0.9300
C37—C38	1.394 (5)	C11—C12	1.369 (7)
C37—C36	1.481 (6)	C11—H11	0.9300
C24—C23	1.369 (6)	C39—H39	0.9300
C24—C25	1.376 (6)	C8—C7	1.500 (6)
C24—H24	0.9300	C8—H8A	0.9700
C18—C17	1.368 (6)	C8—H8B	0.9700
C18—C19	1.380 (6)	C4—C3	1.391 (6)
C18—H18	0.9300	C4—H4	0.9300
C26—C25	1.382 (6)	C25—H25	0.9300
C26—H26	0.9300	C20—H20	0.9300
C34—C33	1.362 (7)	C12—C13	1.362 (6)
C34—H34	0.9300	C13—H13	0.9300
C9—C10	1.392 (5)	C23—H23	0.9300
C9—C14	1.394 (6)	C3—H3	0.9300
			
C22—S2—C28	88.4 (2)	C33—C32—C31	119.9 (4)
C1—S1—C7	88.4 (2)	C33—C32—H32	120.0
C9—O1—C8	118.8 (3)	C31—C32—H32	120.0
C7—N1—C6	110.0 (3)	O3—C29—C28	108.4 (4)
C30—O3—C29	118.7 (3)	O3—C29—H29A	110.0
C28—N2—C27	110.4 (3)	C28—C29—H29A	110.0
C30—C35—C34	118.6 (4)	O3—C29—H29B	110.0
C30—C35—C36	125.0 (4)	C28—C29—H29B	110.0
C34—C35—C36	116.1 (4)	H29A—C29—H29B	108.4
C5—C6—N1	124.7 (4)	C13—C14—C9	119.2 (4)
C5—C6—C1	120.9 (4)	C13—C14—C15	117.1 (4)
N1—C6—C1	114.5 (4)	C9—C14—C15	123.5 (4)
C26—C27—N2	125.8 (4)	C4—C5—C6	118.2 (5)
C26—C27—C22	119.4 (4)	C4—C5—H5	120.9
N2—C27—C22	114.8 (4)	C6—C5—H5	120.9
N2—C28—C29	123.1 (4)	C32—C31—C30	119.5 (4)
N2—C28—S2	116.9 (3)	C32—C31—H31	120.3
C29—C28—S2	120.0 (3)	C30—C31—H31	120.3
C17—C16—C21	118.5 (4)	C23—C22—C27	121.3 (4)
C17—C16—C15	122.5 (4)	C23—C22—S2	129.2 (4)
C21—C16—C15	118.9 (4)	C27—C22—S2	109.5 (3)
C42—C37—C38	118.6 (4)	C37—C42—C41	121.3 (4)
C42—C37—C36	119.5 (4)	C37—C42—H42	119.4
C38—C37—C36	121.9 (4)	C41—C42—H42	119.4
C23—C24—C25	121.1 (5)	C12—C11—C10	118.8 (4)
C23—C24—H24	119.5	C12—C11—H11	120.6
C25—C24—H24	119.5	C10—C11—H11	120.6
C17—C18—C19	119.7 (4)	O4—C36—C37	119.6 (4)
C17—C18—H18	120.1	O4—C36—C35	119.1 (4)
C19—C18—H18	120.1	C37—C36—C35	121.4 (4)
C27—C26—C25	119.0 (4)	F2—C33—C32	119.4 (5)
C27—C26—H26	120.5	F2—C33—C34	118.6 (5)
C25—C26—H26	120.5	C32—C33—C34	122.0 (4)
C33—C34—C35	119.4 (4)	C40—C39—C38	119.4 (4)
C33—C34—H34	120.3	C40—C39—H39	120.3
C35—C34—H34	120.3	C38—C39—H39	120.3
O1—C9—C10	123.1 (4)	O1—C8—C7	106.9 (3)
O1—C9—C14	116.4 (4)	O1—C8—H8A	110.3
C10—C9—C14	120.5 (4)	C7—C8—H8A	110.3
C2—C1—C6	120.5 (4)	O1—C8—H8B	110.3
C2—C1—S1	129.3 (4)	C7—C8—H8B	110.3
C6—C1—S1	110.2 (3)	H8A—C8—H8B	108.6
C20—C19—C18	120.3 (4)	O3—C30—C31	123.3 (4)
C20—C19—Cl1	120.3 (4)	O3—C30—C35	116.1 (4)
C18—C19—Cl1	119.5 (4)	C31—C30—C35	120.6 (4)
C39—C38—C37	120.7 (5)	C5—C4—C3	121.9 (5)
C39—C38—H38	119.7	C5—C4—H4	119.0
C37—C38—H38	119.7	C3—C4—H4	119.0
O2—C15—C16	120.3 (4)	N1—C7—C8	121.9 (4)
O2—C15—C14	118.3 (4)	N1—C7—S1	116.9 (3)
C16—C15—C14	121.4 (4)	C8—C7—S1	121.2 (3)
C20—C21—C16	120.4 (4)	C24—C25—C26	121.0 (4)
C20—C21—H21	119.8	C24—C25—H25	119.5
C16—C21—H21	119.8	C26—C25—H25	119.5
C18—C17—C16	120.9 (4)	C21—C20—C19	120.2 (4)
C18—C17—H17	119.6	C21—C20—H20	119.9
C16—C17—H17	119.6	C19—C20—H20	119.9
C39—C40—C41	121.7 (5)	C13—C12—F1	119.0 (5)
C39—C40—Cl2	119.3 (4)	C13—C12—C11	122.9 (4)
C41—C40—Cl2	118.9 (4)	F1—C12—C11	118.2 (5)
C3—C2—C1	118.2 (5)	C12—C13—C14	119.1 (4)
C3—C2—H2	120.9	C12—C13—H13	120.4
C1—C2—H2	120.9	C14—C13—H13	120.4
C11—C10—C9	119.5 (4)	C24—C23—C22	118.1 (4)
C11—C10—H10	120.3	C24—C23—H23	120.9
C9—C10—H10	120.3	C22—C23—H23	120.9
C40—C41—C42	118.2 (4)	C2—C3—C4	120.4 (5)
C40—C41—H41	120.9	C2—C3—H3	119.8
C42—C41—H41	120.9	C4—C3—H3	119.8
			
C7—N1—C6—C5	178.7 (4)	C26—C27—C22—S2	−179.7 (3)
C7—N1—C6—C1	−0.1 (5)	N2—C27—C22—S2	0.6 (5)
C28—N2—C27—C26	179.8 (4)	C28—S2—C22—C23	177.5 (5)
C28—N2—C27—C22	−0.5 (5)	C28—S2—C22—C27	−0.4 (3)
C27—N2—C28—C29	179.1 (4)	C38—C37—C42—C41	−3.1 (7)
C27—N2—C28—S2	0.2 (5)	C36—C37—C42—C41	178.5 (4)
C22—S2—C28—N2	0.1 (3)	C40—C41—C42—C37	1.2 (7)
C22—S2—C28—C29	−178.8 (4)	C9—C10—C11—C12	1.2 (7)
N2—C27—C26—C25	178.5 (4)	C42—C37—C36—O4	29.3 (6)
C22—C27—C26—C25	−1.2 (6)	C38—C37—C36—O4	−148.9 (4)
C30—C35—C34—C33	−1.4 (7)	C42—C37—C36—C35	−151.1 (4)
C36—C35—C34—C33	−175.2 (4)	C38—C37—C36—C35	30.6 (6)
C8—O1—C9—C10	8.4 (6)	C30—C35—C36—O4	−134.0 (5)
C8—O1—C9—C14	−169.3 (4)	C34—C35—C36—O4	39.3 (6)
C5—C6—C1—C2	0.2 (7)	C30—C35—C36—C37	46.5 (7)
N1—C6—C1—C2	179.1 (4)	C34—C35—C36—C37	−140.2 (4)
C5—C6—C1—S1	−179.3 (3)	C31—C32—C33—F2	−179.9 (4)
N1—C6—C1—S1	−0.4 (5)	C31—C32—C33—C34	−1.1 (8)
C7—S1—C1—C2	−178.8 (5)	C35—C34—C33—F2	−179.5 (4)
C7—S1—C1—C6	0.6 (3)	C35—C34—C33—C32	1.7 (8)
C17—C18—C19—C20	4.0 (7)	C41—C40—C39—C38	−2.4 (7)
C17—C18—C19—Cl1	−177.1 (4)	Cl2—C40—C39—C38	173.6 (3)
C42—C37—C38—C39	2.3 (6)	C37—C38—C39—C40	0.4 (7)
C36—C37—C38—C39	−179.4 (4)	C9—O1—C8—C7	−176.1 (4)
C17—C16—C15—O2	153.1 (4)	C29—O3—C30—C31	−6.9 (6)
C21—C16—C15—O2	−23.8 (6)	C29—O3—C30—C35	171.6 (4)
C17—C16—C15—C14	−26.6 (6)	C32—C31—C30—O3	178.4 (4)
C21—C16—C15—C14	156.5 (4)	C32—C31—C30—C35	−0.1 (7)
C17—C16—C21—C20	1.9 (6)	C34—C35—C30—O3	−177.9 (4)
C15—C16—C21—C20	178.9 (4)	C36—C35—C30—O3	−4.8 (6)
C19—C18—C17—C16	−2.8 (7)	C34—C35—C30—C31	0.7 (6)
C21—C16—C17—C18	−0.1 (6)	C36—C35—C30—C31	173.9 (4)
C15—C16—C17—C18	−177.1 (4)	C6—C5—C4—C3	−0.4 (7)
C6—C1—C2—C3	−0.2 (7)	C6—N1—C7—C8	−178.8 (4)
S1—C1—C2—C3	179.2 (4)	C6—N1—C7—S1	0.6 (5)
O1—C9—C10—C11	−178.5 (4)	O1—C8—C7—N1	176.7 (4)
C14—C9—C10—C11	−0.9 (7)	O1—C8—C7—S1	−2.7 (5)
C39—C40—C41—C42	1.7 (7)	C1—S1—C7—N1	−0.8 (4)
Cl2—C40—C41—C42	−174.4 (4)	C1—S1—C7—C8	178.7 (4)
C30—O3—C29—C28	176.6 (3)	C23—C24—C25—C26	2.0 (8)
N2—C28—C29—O3	−175.2 (4)	C27—C26—C25—C24	−0.9 (7)
S2—C28—C29—O3	3.6 (5)	C16—C21—C20—C19	−0.7 (7)
O1—C9—C14—C13	177.4 (4)	C18—C19—C20—C21	−2.3 (7)
C10—C9—C14—C13	−0.4 (7)	Cl1—C19—C20—C21	178.9 (4)
O1—C9—C14—C15	3.1 (6)	C10—C11—C12—C13	−0.3 (8)
C10—C9—C14—C15	−174.7 (4)	C10—C11—C12—F1	−179.1 (4)
O2—C15—C14—C13	−43.0 (6)	F1—C12—C13—C14	177.7 (4)
C16—C15—C14—C13	136.7 (4)	C11—C12—C13—C14	−1.0 (8)
O2—C15—C14—C9	131.3 (5)	C9—C14—C13—C12	1.4 (7)
C16—C15—C14—C9	−49.0 (6)	C15—C14—C13—C12	176.0 (4)
N1—C6—C5—C4	−178.7 (4)	C25—C24—C23—C22	−0.9 (7)
C1—C6—C5—C4	0.1 (7)	C27—C22—C23—C24	−1.1 (7)
C33—C32—C31—C30	0.3 (7)	S2—C22—C23—C24	−178.9 (4)
C26—C27—C22—C23	2.2 (6)	C1—C2—C3—C4	−0.1 (8)
N2—C27—C22—C23	−177.5 (4)	C5—C4—C3—C2	0.4 (8)

### Hydrogen-bond geometry (Å, º)

Cg1 and Cg2 are the centroids of the thiazole rings S1/C1/C6/N1/C7 and S2/C22/C27/N2/C28, respectively.

**Table:**

*D*—H···*A*	*D*—H	H···*A*	*D*···*A*	*D*—H···*A*
C3—H3···F1^i^	0.93	2.52	3.091 (6)	120
C5—H5···O2^ii^	0.93	2.46	3.340 (5)	158
C26—H26···O4^iii^	0.93	2.51	3.369 (5)	154
C18—H18···*Cg*1^iv^	0.93	2.83	3.686 (5)	154
C39—H39···*Cg*2^v^	0.93	2.82	3.619 (5)	145

Symmetry codes: (i) −*x*+1/2, *y*+1/2, *z*−1/2; (ii) *x*−1/2, −*y*+3/2, *z*; (iii) *x*+1/2, −*y*+1/2, *z*; (iv) *x*, *y*−1, *z*; (v) *x*, *y*+1, *z*.
